# Mitochondria, Oxidative Stress, cAMP Signalling and Apoptosis: A Crossroads in Lymphocytes of Multiple Sclerosis, a Possible Role of Nutraceutics

**DOI:** 10.3390/antiox10010021

**Published:** 2020-12-28

**Authors:** Anna Signorile, Anna Ferretta, Maddalena Ruggieri, Damiano Paolicelli, Paolo Lattanzio, Maria Trojano, Domenico De Rasmo

**Affiliations:** 1 Department of Basic Medical Sciences, Neurosciences and Sense Organs, University of Bari Aldo Moro, 70124 Bari, Italy; maddalena.ruggieri@uniba.it (M.R.); damiano.paolicelli@uniba.it (D.P.); maria.trojano@uniba.it (M.T.); 2 CNR-Institute of Biomembranes, Bioenergetics and Molecular Biotechnologies, 70126 Bari, Italy; a.ferretta@ibiom.cnr.it (A.F.); p.lattanzio@ibiom.cnr.it (P.L.)

**Keywords:** multiple sclerosis, mitochondria, cAMP, apoptosis, oxidative stress, nutraceutics

## Abstract

Multiple sclerosis (MS) is a complex inflammatory and neurodegenerative chronic disease that involves the immune and central nervous systems (CNS). The pathogenesis involves the loss of blood–brain barrier integrity, resulting in the invasion of lymphocytes into the CNS with consequent tissue damage. The MS etiology is probably a combination of immunological, genetic, and environmental factors. It has been proposed that T lymphocytes have a main role in the onset and propagation of MS, leading to the inflammation of white matter and myelin sheath destruction. Cyclic AMP (cAMP), mitochondrial dysfunction, and oxidative stress exert a role in the alteration of T lymphocytes homeostasis and are involved in the apoptosis resistance of immune cells with the consequent development of autoimmune diseases. The defective apoptosis of autoreactive lymphocytes in patients with MS, allows these cells to perpetuate, within the CNS, a continuous cycle of inflammation. In this review, we discuss the involvement in MS of cAMP pathway, mitochondria, reactive oxygen species (ROS), apoptosis, and their interaction in the alteration of T lymphocytes homeostasis. In addition, we discuss a series of nutraceutical compounds that could influence these aspects.

## 1. Introduction

Multiple sclerosis (MS) is an inflammatory and neurodegenerative chronic disease that involves immune and central nervous system (CNS) [1,2]. MS presents different clinical phenotypes: relapsing-remitting (RR-MS), which is the prevalent form and it characterized by various attacks with neurologic symptoms, such as altered sensation, weakness, impairment of visual acuity, or double vision, balance disturbance, followed by periods of partial or complete recovery. The majority of untreated RR-MS patients do eventually progress into secondary progressive (SP-MS) course caractherized by a history of a gradual disability progression, independent of relapses; primary progressive (PP-MS) course, characterized by worsening of neurological functions from the onset of the disease [3]. As few as 5% of MS patients present progressive neurological deficits with acute attacks with or without recovery, named progressive-relapsing MS (PR-MS) [4,5]. In 2013, these originally established MS subtypes were revised to review potential imaging and biological correlates and to reflect further MS clinical aspects. Thus, new phenotypes, radiologically isolated syndrome, and clinically isolated syndrome were considered in addition with two new subtypes: activity and progression applied to RR and progressive MS phenotypes [6].

The pathogenesis of MS involves the loss of blood-brain barrier (BBB) integrity resulting in the invasion of lymphocytes into CNS, with the consequent tissue damage [6]. Despite immunology, cell biology, and genetic knowledge [1,2,3,6], the etiology of MS remains unknown, but it is probably an interaction among autoimmune, genetic, and environmental factors [7,8,9,10,11] which can affect a disturbed immune response. T lymphocytes play a key role in the initiation and propagation of MS, in fact, these cells, once infiltrated in the CNS, release a large amount of pro-inflammatory cytokines that activate macrophages, leading to the inflammation [6,7,8,9,10,11,12], and consequently to myelin sheath destruction [6] in both white and grey matter regions [12]. Furthermore, the stimulation of T helper (Th) lymphocytes (presumably Th1) and their lymphokines synthesis, such as interferon-γ (IFN-γ) and interleukin-2 (IL-2), induces B lymphocytes to transform into plasma cells. B cells represent almost 40% of all CNS infiltrating lymphocytes and they can costimulate activated T cells, producing autoantibodies against components of neurons, i.e., axon and myelin. The response of immune system is regulated by subpopulations of T lymphocytes, including Th (CD4^+^), cytotoxic T cells (CD8^+^), regulatory T cells (Tregs) (CD4^+^/CD25^+^), and natural killer cells (CD16^+^/CD56^+^) [13].

Many cellular processes participate in the activation of lymphocytes, among others cyclic AMP (cAMP) [14], mitochondrial dysfunction [15] and oxidative stress (OS) [16] play a crucial role and have a further regulatory effect on many disease aspects [14,15,16,17,18]. cAMP is the most studied second messenger in the context of T lymphocyte activation and proliferation [19]. It has been reported that the increase of cAMP level attenuates the T lymphocyte-mediated production of pro-inflammatory cytokines such as IFN-γ and interleukin (IL)-1b (IL-1b) suggesting that a decrease of cAMP level is required for T cell activation [14,20,21]. The activation of immune cells, especially T cells, is also related to OS [15] and the peripheral blood mononuclear cells (PBMCs) of MS patients show an impaired redox status associated with a metabolic reprogramming that includes mitochondrial alterations [22,23,24,25].

Several mechanisms participate in the maintenance of the immune homeostasis preventing autoimmune disease developments. In this regard, the apoptosis is an important process that removes potentially pathogenic autoreactive lymphocytes, limiting the tissue damage caused by immune response [26,27]. Although the OS is an activator of apoptotic process, the lymphocytes in MS appear to be resistant to apoptotic stimuli. The defective apoptosis of autoreactive lymphocytes in patients with MS, allows these cells to propagate, within the CNS, a continuous cycle of inflammation [28,29]. In particular, in CD4^+^ T lymphocytes of MS patients, the impairment of mitochondria-mediated apoptosis and a reduction of mitochondrial respiration are reported [30,31].

Mitochondria are important organelles for both cell death and life, and they are a major source of reactive oxygen species (ROS) production. At the same time, mitochondria are responsive to OS and other cellular signalling and are critical in modulating apoptosis. In this review, we will discuss the involvement of mitochondria, ROS production, apoptosis, and cAMP pathway, and how they can mutually modulate each other in the alteration of T lymphocytes homeostasis in MS. In addition, we discuss a series of nutraceutical compounds that could influence all these aspects.

## 2. Mitochondrial Alterations of T Lymphocytes in MS

A central part of the T cell activation is the change of metabolism in the cell. Proliferating T cells need more ATP for enhanced activity and acquire anabolic capacity to sustain the growth. Also, the most dramatic change in T cell metabolism, upon activation, is a marked increase in glucose metabolism through the increase localization of glucose transporter 1 (GLUT1) to the plasma membrane and the enhance of glycolytic enzyme activities [20,32]. Activated T cells take up large amounts of glucose associated with lactate production. Indeed, activated T cells presented primarily glycolytic despite oxygen supply (aerobic glycolysis) [33,34]. However, while glycolysis is a rapid source of ATP and it can lead the pentose phosphate pathway to generate NADPH and nucleotides, it could be insufficient to completely sustain cell proliferation. Interestingly, upon activation, T cells also augment glutaminolysis process, that producing α-ketoglutarate, supplies the mitochondrial tricarboxylic acid (TCA) [35,36]. This is important in proliferating cells in which the intermediates of TCA cycle are continually depleted for their use in biosynthetic pathways [36,37]. Thus, mitochondrial metabolism plays a crucial role in T cell activation. Mitochondrial metabolism is a critical component of T cell activation also through mitochondrial-dependent ROS production [34,38] that, in turn, is also involved in the activation of a biosynthetic pathway of nucleic acid and T-cell proliferation [33].

One of first evidence of mitochondrial alteration in T lymphocytes of MS comes from the electron microscopy observation of abnormal mitochondria in shape and size and very often with thickened cristae [39]. Change in mitochondrial structure are associated, indeed, with changes in mitochondrial metabolism and mitochondrial apoptosis. Nowadays, decreased complex I and complex IV activities have been reported in lymphocytes of subjects affected by RR-MS with respect to those of control subjects associated with a decrease of their constituent subunits [40]. The reduced activity and protein expression of constituent subunits is also reported for complexes III and V of oxidative phosphorylation system (OXPHOS) in PBMC of RR-MS patients [24]. Also, a reduction of mitochondrial membrane potential, according to decrease of respiratory electron flux, has been found in lymphocytes of a cohort of 65 MS patients [41]. Very interesting, the decrease of mitochondrial respiratory chain activity and mitochondrial membrane potential are associated with disease severity [24,41]. Moreover, mitochondrial membrane potential reduction can be ascribed to a mitochondrial decoupling. In fact, studies reported a decrease of the ratio of mitochondrial ATP over glycolytic ATP in lymphocytes of MS subjects depending on both mitochondrial uncoupling, as shown by reduction of oxygen consumption in the presence of ADP, and increase activity of key enzymes of glycolysis such as hexokinase and phosphofructo kinase I [40]. According to the increase of glycolytic flux, an increase of GLUT1 expression has been found as well as an increase of secreted lactate [40]. In agreement with these results, another work reported a decrease of mitochondrial respiration activity but also a decrease of glycolytic flux [29].

**Figure 1:**
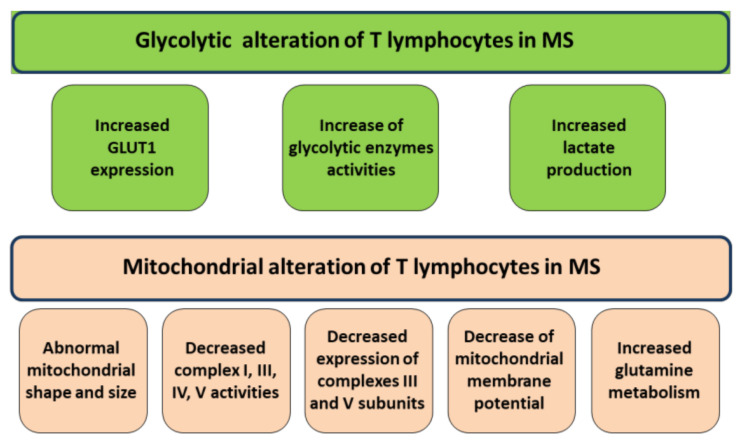
Metabolic alterations of T lymphocytes in multiple sclerosis (MS). Activated T cells metabolize large amounts of glucose associated with lactate production by increasing glucose transporter 1 (GLUT1) expression at the plasma membrane [40]. In addition, to sustain cell proliferation, T cells also augments glutamine metabolism [35]. Mitochondria of T lymphocytes of MS patients show abnormal structure, decrease of oxidative phosphorylation system (OXPHOS) in term of subunit expression and complex activities and decrease of mitochondrial membrane potential [24,39,40,41].

So far, a reduced activity of mitochondrial enzymes and bioenergetics decoupling of mitochondria in term of efficiency to produce ATP has been observed and confirmed, in MS patients’ lymphocytes (Figure 1), by using different experimental approaches and conditions [24,40,41]. More reports show an increase of glycolytic flux in MS lymphocytes. However, another work observed a reduced glycolysis associated with reduced expression of GLUT1 [31] (Figure 1).

## 3. Oxidative Stress in Lymphocytes of MS

**Figure 2:**
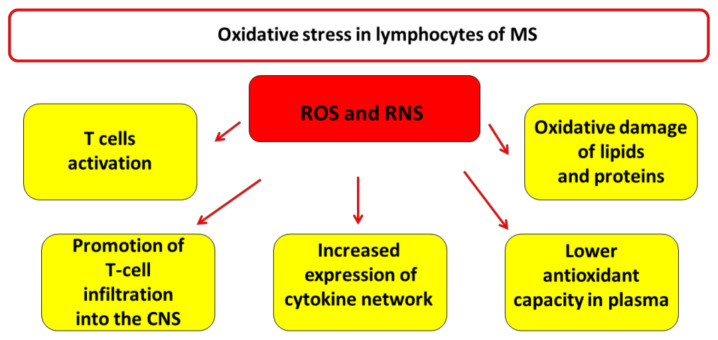
Impact of oxidative stress in T lymphocytes of multiple sclerosis (MS). Oxidative stress (OS) plays an important role in the pathogenesis of MS. The major radical species are represented by reactive oxygen species (ROS) and reactive nitrogen species (RNS). OS is involved in several characteristic process of MS such as the activation of immune cells [15], especially T cells, promotion of T-cell migration and infiltration into the central nervous system (CNS) [47] and the increased expression of the cytokine network [50]. The plasma of MS patients shows a lower antioxidant capacity and higher levels of lipids and proteins oxidative damage [44,45,46,47].

Oxidative stress condition is characterized by the imbalance between the production of free radicals and their counteraction by antioxidant defenses [42]. An increasing number of studies has shown that OS plays an important role in the pathogenesis of MS [5,6,22,23,43,44]. OS, associated with inflammation and neuronal damage, results in the oxidation of cellular components, such as proteins, lipids, and nucleic acids potentiates leading to a vicious cycle that can accelerate the progression of the disease [5,44,45,46,47]. OS is involved in several characteristic process of MS such as the activation of immune cells, especially T cells [15], the loss of BBB selectivity [48], promotion of T-cell migration and infiltration into the CNS [49] and the increased expression of the cytokine network [50] (Figure 2). The major radical species are represented by ROS and reactive nitrogen species (RNS) that are produced by specific brain cells such as astrocytes, activated macrophages, and microglia in the CNS of MS patients.

ROS are highly reactive molecules due to the presence of unpaired electrons and they can generate new free radicals reacting with other biological molecules. Many different forms of ROS exist, anion superoxide (O_2_^−^), hydroxyl radical (OH∙) and hydrogen peroxide (H_2_O_2_). Anion superoxide can be produced by NAD(P)H oxidase and as a by-product of oxidative phosphorylation. Anion superoxide has a short life span, and it can be rapidly converted by superoxide dismutases (SODs) into hydrogen peroxide or into other ROS [51]. Hydrogen peroxide is relatively stable and permeates cellular membranes generating hydroxyl radicals by Fenton reaction. Furthermore, ROS are produced in large amount by immune cells to kill pathogens and facilitate phagocytosis [52]. However, in several neurological diseases, including Huntington’s disease, Parkinson’s disease, Alzheimer’s disease, and MS, the production of ROS overwhelms the cellular antioxidant capacity contributing to cellular injury [53,54].

In macrophages and microglial cells, the inflammatory cytokines such as tumor necrosis α (TNF-α) and IFN-γ inducing nitric oxide synthase (NOS), are responsible for the production of nitric oxide (NO). There are three major NOS isoforms: neuronal (nNOS), endothelial (eNOS), and inducible (iNOS). In an experimental autoimmune encephalomyelitis (EAE) animal model, an increase expression of iNOS mRNA and protein has been associated with the severity of clinical symptoms [55,56,57]. In addition, acting as a vasodilator, NO increases BBB permeability allowing inflammatory cells to infiltrate the CNS [58]. NO can react with superoxide anions to generate RNS such as peroxynitrite (ONOO−), which is a very dangerous oxidant associated with neuronal loss and is considered as pathogenic agent in MS [59]. Although the literature suggests that OS may be involved in the pathogenesis of MS in which activated microglia and macrophages are major cell source of ROS, few evidences are reported on OS in peripheral T lymphocytes.

Mitochondria are one of the major sources of ROS. They normally produce O_2−_ at point of electron leakage in the electron transfer chain essentially at complex I and complex III. Mitochondria dysfunctions can result in an excessive O_2_^−^ production that induces overexpression of the mitochondrial manganese superoxide dismutase (Mn-SOD), which increases production of H_2_O_2_ [60]. H_2_O_2_ is reduced to water by glutathione peroxidase in both mitochondrial matrix and cytosol. H_2_O_2_, which diffuses to peroxisomes, is converted to water and oxygen by catalase [60]. Data reported that the plasma of MS patients show a lower antioxidant capacity [61] and higher levels of oxidative damage to lipid and proteins [61,62]. Our study conducted in PBMCs from 15 MS patients and 15 healthy subjects showed increased level of H_2_O_2_ in MS with respect to healthy subjects [25] detected spectrofluorimetrically by dichlorodihydrofluorescin (DCFDA) probe. Interesting, the increased level of ROS production correlated with deregulation of some proteins involved in the mitochondrial dynamics and apoptosis [25]. Another report, carried out in PBMC of 34 MS and 24 healthy subjects, showed a higher concentration of anion superoxide in MS evaluated by flow cytometry, associated with a decrease in the protein content of the mitochondrial OXPHOS (complexes I–V) [24].

Mechanistically, the increase of ROS production can come from two hypotheses. The first is the stimulation of TCA cycle in glycolytic lymphocytes, resulting in an increase of mitochondrial NADH to a level greater than that needed to sustain mitochondrial respiration, that associated with the reduced expression of mitochondrial respiratory chain subunits [24,33] saturates the electron transport chain with consequent increased ROS production. The second is the decoupling of mitochondrial respiratory chain as already reported in lymphocytes of MS patients [40] that can result in an increased production of anion superoxide. These findings support the mitochondrial involvement in lymphocyte ROS production in MS. However, the OS in peripheral lymphocytes and its contribution to the disease remain to be analyzed more in depth.

## 4. Role of cAMP Signaling in T Lymphocyte of MS

In the context of T lymphocyte differentiation, proliferation and activation, cAMP is the most studied second messenger and it has well recognized as potent negative regulators of immune function of T cells [14,20,21]. In mammalian cells, cAMP is produced by a family of transmembrane adenylyl cyclases (tmACs) and by the soluble adenylyl cyclase (sAC), that generates cAMP pools in various intracellular compartments, including mitochondria. tmAC is activated by β-adrenergic receptor stimulation, while sAC is modulated by intracellular stimuli, such as calcium, bicarbonate, and ATP [63]. cAMP level is also controlled by a super family of phosphodiesterases (PDEs) enzymes that hydrolyze and inactivate cAMP signalling. cAMP can act through different effectors, protein chinase A (PKA) (isoform I and II, PKA I and PKA II, respectively) and/or exchange protein directly activated by cAMP (EPAC). cAMP/PKA signalling regulates T cell function at the level of transcription factors, members of mitogen-activated protein kinase (MAPK) pathway, phospholipases 8PLs, ras homolog (RhoA) and proteins implicated in the control of cell cycle progression [64]. In the T cells, 80% of the total PKA activity is associated with PKA I, whereas approximatively 10–20% is accounted for by PKA II [19]. Specific involvement of the isoforms I and II of PKA can give a different role of cAMP in cell fate. For example, specific cAMP analogues for the isoform I inhibit natural killer cell-mediated cytotoxicity [65].

Many studies report the contribution of elevation of cAMP in the immunosuppression. Indeed, increased cAMP level in response to prostaglandin E2 (PGE2) and other agents has been reported to cause immunosuppression [19]. Interestingly, activation of the cAMP/PKA pathway has been implicated in the T cell dysfunction associated with human immunodeficiency virus (HIV) infection and a subset of common variable immunodeficiency [66]. Conversely, the inhibition of PKA I is reduced in patients with the autoimmune disease systemic lupus erythematosus [67]. It is reported that lipoic acid attenuates inflammation with reduction of IL-6 via cAMP and protein kinase signaling [68]. Data from EAE models indicated that lipoic acid may be effective as a treatment for MS by stimulating the production of cAMP in T-cell enriched peripheral blood mononuclear cells [69]. Moreover, the total cAMP level in PBMC was found reduced in RR-MS with respect to healthy subjects [69]. This is consistent with other reports in which the use of the cAMP elevating agents, such as forskolin, dibutyryl cyclic AMP (dbcAMP), and cholera toxin, reduces Il-2 level in murine T cells [70]. Moreover, in experimental models of EAE, dbcAMP inhibits the progression of disease, improves spatial memory retention in male rats, attenuates demyelination and enhances endogenous neural stem cell recruitment, [71,72,73].

**Figure 3:**
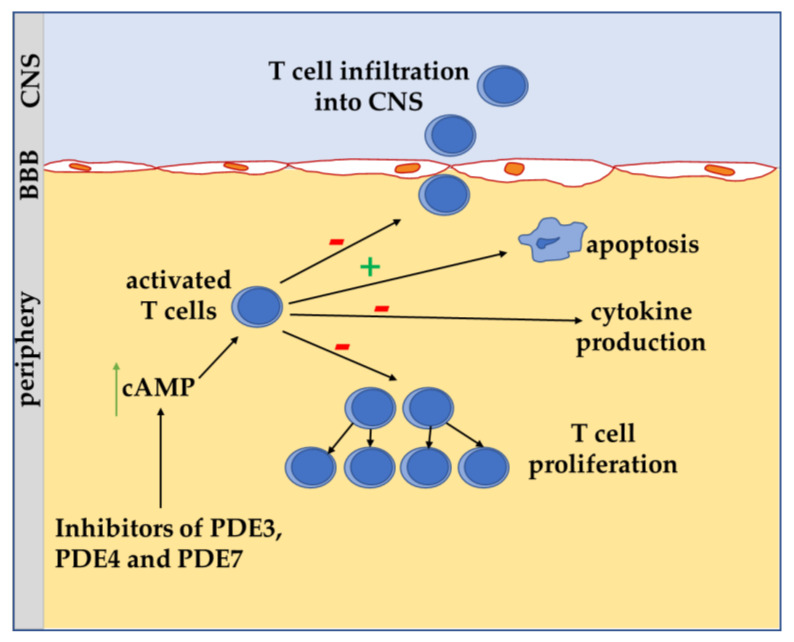
Schematic view of impact of cAMP on T cells in multiple sclerosis. Inhibition of phosphodiesterase 3 (PDE3), 4 (PDE4) and 7 (PDE7) results in an increase of cAMP level that can lead to augmented T cell apoptosis, reduction of infiltration by crossing the blood-brain barrier (BBB) into central nervous system (CNS), decrease of cytokine production and T cell proliferation [19,66,68,70,74,75,79,80].

Thus, the general observation on T cells is that increasing cAMP levels, reducing the production of pro-inflammatory cytokines (e.g., IFN-γ, TNF-α, and IL-1β), T cell proliferation and T cell activation attenuates the T lymphocyte mediated immune response [66]. Conversely, consistent with these results, a decrease of cAMP primes T cell activation [69]. Furthermore, increased cAMP level is important for the development of Tregs to maintain immunological homeostasis by suppressing the innate immune responses [66]. Evidence indicates an important role of PDEs in the modulating cAMP level in MS and thus T cell activation and proliferation. It has been reported that anti CD3/CD28 stimulation to activate naïve CD4^+^ T cells resulted in increased enzymatic activity of PDE7 [74]. Accordingly, in EAE mice, the use of the PDE7 inhibitor, named TC3.6, increased the mRNA level of forkhead box P3 (FOXP3) protein, a master regulator of Treg formation, the production of anti-inflammatory cytokine IL-10, and decreased the level of pro-inflammatory cytokine IL-17, T cell proliferation and T cell infiltration into CNS [74]. However, PDE7A knockout mice failed to alter T cell activation and/or cytokine production [75]. In the context of pro-inflammatory processes, PDE4 is the most studied cAMP-specific PDE. As observed for PDE7, in EAE mice, the inhibition with rolipram of PDE4 decreased T cell proliferation and reduced the production of TNF-α and IL-17 while increasing the production of IL-10 [21,74]. In addition, rolipram decreased the number of perivascular inflammatory infiltrates associated with a reduction of clinical symptoms [21,74]. Furthermore, in activated human CD4^+^ T cells, knockdown by siRNA of PDE4D reduced their proliferation rate and reduced the secretion of IFN-γ [76]. In EAE mice, mRNA level of the PDE4B2 isoform has been found increased in infiltrating T cells in the CNS [77]. The increased PDE4B2 positively correlated with FOXP3 and transforming growth factor beta (TGF-β) mRNA levels, supporting a regulatory role for PDE4B2 in Treg modulation [77,78]. As for PDE7 and PDE4, the dual substrate PDE3 inhibitor, cilostazol, has been shown to ameliorate T cell response in EAE mice by reducing IFN-γ production and lymphocytic proliferation in the CNS [79]. Summarizing, increasing cAMP by inhibiting specific PDEs could be considered as a potential therapeutic strategy to reduce the activation of T cell and the production of proinflammatory cytokines [21,74] (Figure 3). It should be noted that inhibitors of PDE3, 4, and 7 induce apoptosis in leukemic cells [74,80].

## 5. Apoptosis and Lymphocytes in MS

Programmed cell death (apoptosis) is a key anti-autoimmune mechanism that removes pathogenic autoreactive lymphocytes from the circulation and tissues, and controls the immune-mediated tissue damage [81]. More evidence suggests that apoptotic deletion of autoreactive lymphocytes is defective in patients with MS, and the relevance of this process in MS pathogenesis is confirmed considering that some disease modifying therapies, that is known to have beneficial effects in the disease, act by pushing T lymphocytes towards apoptosis [28,29,35,82]. Both peripheral and intrathecal lymphocytes from MS patients show reduced susceptibility to extrinsic and intrinsic cell death [29,83]. Indeed, the apoptosis is mediated through two main pathways: (i) extrinsic or death receptor-induced mechanism, which includes the surface receptor Fas (CD95) and other members of the tumor necrosis factor receptor family; and (ii) intrinsic or mitochondria-mediated pathway. The cause of lymphocyte resistance to cell death in MS is not fully understood but it appears to involve apoptotic defects at multiple cellular levels, as, for instance, the elevated expression of antiapoptotic factors [84].

Involvement of impairment of mitochondria-mediated apoptotic deletion has been reported in CD4^+^ T lymphocytes of PP-MS patients [30]. Evidence has been presented for a role of T CD4^+^ cells expressing the chemokine receptors CCR5 and CXCR3 in the trafficking of activated memory T cells of Th1 phenotype into the CNS of MS patients [85]. It has been observed that CD4^+^CCR5^+^ T cells from MS patients were more resistant to Fas-induced apoptosis compared with healthy subjects [30]. In this study, it has been shown that defects in apoptosis were more significant in patients with PPMS which also presented a reduction of mitochondrial membrane potential rising the implication of the intrinsic mitochondrial apoptotic pathway in the Fas-mediated apoptosis of CD4^+^CCR5^+^T cells [30]. A cross-talk between the extrinsic Fas-mediated and intrinsic pathways exists, indeed, through the Bcl-2 family member Bid, which, after cleavage by caspase-8, translocates to mitochondria where it promotes the cytochrome c release [86].

Mitochondria-mediated mechanism of apoptosis involves Bcl-2 proteins, that have anti-apoptotic function (Bcl-2 like), Bax proteins (Bax-like) and other proteins such as Bad, Bak and Bim, that have pro-apoptotic function [87]. The Bcl-2 gene has an anti-apoptotic activity in different cell types, including lymphocytes [88], in fact, upregulation of Bcl-2 proteins may cause systemic autoimmune disease as result of activated T cells accumulation [89]. Histopathologic studies have observed in MS plaques high level of lymphocytes expressing Bcl-2 [90]. However, other studies failed to find a significant Bcl-2 upregulation in intrathecal or peripheral lymphocytes from MS patients [91,92,93]. Other mitochondria parameters, such as fusion and fission, and mitochondria cristae architecture are involved in mitochondrial apoptosis [94,95,96]. Several mitochondrial proteins have been found to play a key role in mitochondria-mediated apoptosis in MS lymphocytes (see Table 1). Among others, optic atrophy 1 (OPA1) protein is a mitochondrial dynamin like GTPase that regulates mitochondrial fusion, the mitochondrial respiratory chain complex stability, the maintenance of mitochondrial cristae architecture and pro-apoptotic cytochrome c release [97]. Interestingly, it is observed that OPA1 gene mutations, resulting in autosomal dominant optic atrophy (ADOA), are correlated with multiple sclerosis-like disorders [98]. OPA1 undergoes constitutive proteolytic processing resulting in un-cleaved long OPA1 (L-OPA) and in a cleaved short OPA1 (S-OPA) forms. The balance between L-OPA1 and S-OPA1 influences the apoptosis susceptibility. Many stress conditions, such as apoptotic stimuli, trigger L-OPA1 conversion into S-OPA1 [95]. Recently it has been shown in PBMC of MS patients a different proteolytic processing of OPA1 compared with healthy controls [25]. The activity and processing of OPA1 are controlled by mitochondrial proteases, OMA1 and YMEL1 and regulated by post-translational modification such as sirtuin 3 (SIRT3)-dependent acetylation status [95,99] and OS [95,100]. OMA1-dependent degradation of OPA1 is a cellular response to OS, in fact, although OMA1 is constitutively active, it shows an enhanced activity in response to OS [100]. However, PBMCs of MS patients present an increase of ROS level that does not lead to the increase of the stress-induced active form of OMA1, but rather to the accumulation of the inactive form of OMA1 [25]. SIRT3 has a crucial role in many OS-mediated cellular responses [100], in the controlling of bioenergetics and antioxidant defense of mitochondria under OS [101]. OS regulates the level of SIRT3 protein that, in turn, modulates OPA1 acetylation/processing evolving in the regulation of mitochondrial apoptosis [95]. In particular, sustained SIRT3 protein level supports apoptosis resistance, while a reduced level promotes cell death [95]. Data showed that, despite the augmented ROS levels in MS, no change has been detected in the level of SIRT3 protein in MS lymphocytes compared to healthy control samples [25]. Although the level of SIRT3 protein was unaffected, this could be interpreted as a failure of reaction to OS of lymphocytes of MS patients. The data on OMA1 and SIRT3 suggest a deregulated stress response mechanism in PBMCs of MS patients that, in turn, alters OPA1 proteolytic processing. Furthermore, OPA1 stability also is under the control of prohibitin 2 (PHB2), a chaperone like protein, localized in nucleus, plasma membrane and mitochondria [102]. In inner membrane of mitochondria, PHB2 forms with prohibitin 1 (PHB1) a large membrane-bound complex [103,104] that is required for OPA1 stability [103,104]. Deletion of PHB2 results in decreased cellular proliferation, aberrant morphogenesis of mitochondrial cristae, and apoptosis, while PHB2 over-expression is shown to protect from apoptosis [104,105]. Evidence indicates that mitochondrial PHB2 is over-expressed in lymphocytes of MS patients [25,106,107] thus representing another element of resistance to apoptosis. Moreover, PHB2 is involved in the mechanism of mitophagy (selective autophagy) by functioning as an autophagosome formation receptor [108]. Factors involved in the process of autophagy have been implicated in neurodegenerative diseases and molecules involved in autophagy have also been found to impinge with T cells homeostasis [109]. In particular, autophagy-related gene-5 (Atg5)-deficient T lymphocytes display a reduced number in vivo, increased T cell apoptosis, and an incapability to undergo T cell receptor-induced proliferation [110]. Other studies have shown that Atg5 post-translational cleavage can also induce T cells apoptosis, on the contrary, an important change in Atg5 T cells, may be associated with the increase of T cell survival and/or the promotion of T cell proliferation during active disease [111]. It has reported that the upregulation and post-translational modification of Atg5 in autoreactive T cells of MS patients and in the mouse model of EAE correlated with viability of T cells and may contribute to inflammatory demyelination in MS [112].

## 6. The cAMP Signalling in the Regulation of Mitochondrial Activity, ROS Balance and T Cell Proliferation: Possible Implications of Some Nutraceutics

**Figure 4:**
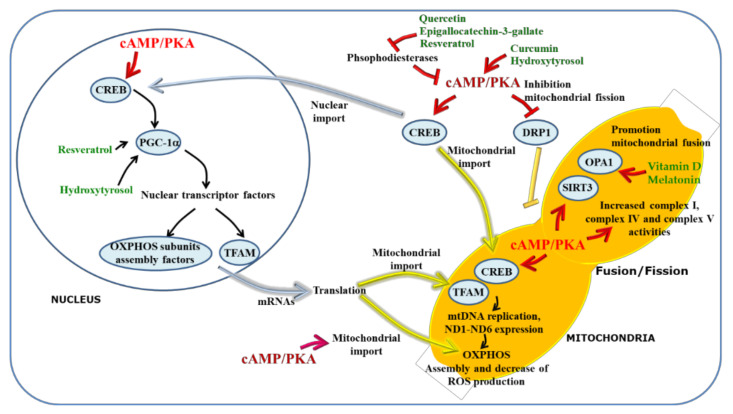
Control by the cAMP pathway of mitochondria. PKA-dependent phosphorylation of CREB activates the PGC-1 alpha transcription cascade promoting the expression of nuclear genes coding for OXPHOS subunits and mitochondrial transcription factor (TFAM) [115]. In addition to the nucleus, CREB resides inside mitochondria, where it binds to the D-loop of mitochondrial DNA inducing expression of structural OXPHOS components [116,117]. PKA-dependent phosphorylation of the NDUFS4 subunit increases its import into mitochondria and assembly in complex I [118,119,120]. sAC-dependent cAMP production also promotes the complex I, IV and V activities [121,122,123,124]. PKA-dependent phosphorylation of DRP1, inhibits its pro-fission activity, thus promoting mitochondrial fusion and respiration [125,126]. sAC-dependent cAMP stabilizes SIRT3 expression and OPA1 proteolytic processing promoting mitochondrial fusion and resistance to apoptosis [95]. The nutraceutics are shown in green, closed to the target molecules, for details see the text. The cAMP/PKA pathway is shown in red, the nuclear protein transport in bluette and the mitochondrial protein import in yellow.

Summarizing, reduced mitochondrial respiration and mitochondrial membrane potential, increased ROS production and defected mitochondrial-mediated apoptosis have been observed in T cells of MS patients. Moreover, reduction of T lymphocyte mediated immune response, T cell proliferation and T cell activation in response to elevation of cAMP levels [19,21,60] as well as a reduction of cAMP level in RR-MS lymphocytes [69] have been observed. Several studies have reported that an antioxidant therapy has beneficial effects in vivo and in vitro animal models for MS. In the context of antioxidant therapy, due to their own antioxidant properties, the use of nutraceutics in neurodegenerative, inflammatory, and metabolic diseases is in constant expansion in order to ameliorate or prevent aging-related diseases [113]. These molecules are easily extracted from plants or derived from fermentation in bioreactors [113]. Among people with MS, nutraceutics are commonly used, often concomitantly with conventional treatment [114]. We would like to highlight that cAMP strongly affects both mitochondrial functions, ROS production, and mitochondria-mediated apoptosis and that some nutraceutical compounds, beyond the own antioxidant effect and inhibition of inflammation, can act by targeting mitochondria, apoptosis, and cAMP signaling (Figure 4).

### 6.1. cAMP, Mitochondria and ROS Balance

Defects of complex I and IV activities and their protein expression have been observed in MS lymphocytes. The complex I of mitochondrial respiratory chain is the major source of ROS and it is strongly regulated by cAMP pathway (Figure 4). Several studies have shown that cAMP/PKA signaling has a positive regulatory effect on ROS balance and complex I. In fact, the induced increase of cAMP rescues the activity of complex I and the increase of ROS levels occuring in G0 phase in murine and human cells and in oxidatively damaged cells [118,127,128]. Many reports showed that the increase of cAMP level, by stimulation of tmAC in mammalian cell cultures, increases the activity of complex I and decreases the complex I-dependent ROS production by promoting the PKA-dependent phosphorylation, mitochondrial import, and assembly in complex I of NDUFS4 subunits [118,119,120]. Other works reported the involvement of cAMP/PKA pathway in the regulation of complex IV activity [129]. Moreover, the cAMP affects the mitochondrial biogenesis via cAMP response element binding (CREB) protein. Upon phosphorylation by PKA, the CREB protein activates the expression of peroxisome proliferator-activated receptor gamma coactivator 1-alpha (PGC-1α), which in turn induces the expression of downstream nuclear and mitochondrial transcription factors, such as TFAM [115] (Figure 4).

Beyond to reside in the nucleus, CREB has also been reported to reside in mitochondria, where it, by binding to the D-loop of mitochondrial DNA [116], induces the expression of OXPHOS subunits, in particular the seven genes of complex I [117]. Mitochondrial pool of cAMP, resulting from mitochondrially localized sAC, increases the activity of cytochrome—*c* oxidase [121], the ATP production [122], the complex I activity and the turnover of its nuclear-encoded subunits [123] and controls the structural organization and functional activity of FoF1 ATP synthase [124].

As for mitochondrial respiratory chain activity, mitochondrial size, shape (dynamics) and cristae structure, and mitochondrial shaping protein (OPA1) have been found altered in MS lymphocytes. The mitochondrial dynamics and structure depend essentially on dynamin-related protein 1 (DRP1) proteins and OPA1, influencing several processes of the cell such as apoptosis [125]. Mitochondria fission is mainly controlled by the dynamin-like GTPase, DRP1, which is recruited to the surface of mitochondria and assembled around the constriction points of the dividing mitochondria into a multimeric ring-like structure [126]. PKA-dependent phosphorylation of DRP1 blocks its translocation to the surface of mitochondria, resulting in inhibition of fission and so in a mitochondrial elongation, which promotes cell survival. DRP1 dephosphorylation facilitates its translocation to mitochondrial surface promoting fission, apoptosis, and autophagy [126,130]. In addition, mitochondrially produced cAMP regulates OPA1 stability. In particular, mitochondrial cAMP reduction causes OPA1 proteolytic processing by inhibiting SIRT3-dependent protein deacetylation, resulting in an anti-fusion event and apoptosis [95]. On the contrary, increase in mitochondrial cAMP enhances SIRT3 protein level and promotes fusion events that protect myoblasts from ROS-dependent apoptosis [95]. In addition to DRP1, PKA phosphorylates mitofusin 2 (Mfn2) [131] (Figure 4).

In the context of mitochondrial cAMP, it is worth mentioning that the treatment with cysteamine of the EAE mice model ameliorated disease severity. Cysteamine has been also found to increase specifically mitochondrial cAMP resulting in increase of mitochondrial respiration, membrane potential, cristae structure and fusion process [132,133].

cAMP, acting through different effectors, PKA and/or EPAC, could determine a pro-apoptotic or anti-apoptotic response [134]. In addition, a specific involvement of one of the two isoforms of PKA (I and II) can impinge with the role of cAMP in cell death. For example, analogues of cAMP specific for the isoform I inhibit natural killer cell-mediated cytotoxicity [65]. Instead, expression of PKA isoform II can modulate apoptosis of fibroblasts [135].

### 6.2. Nutraceutics

**Figure 5:**
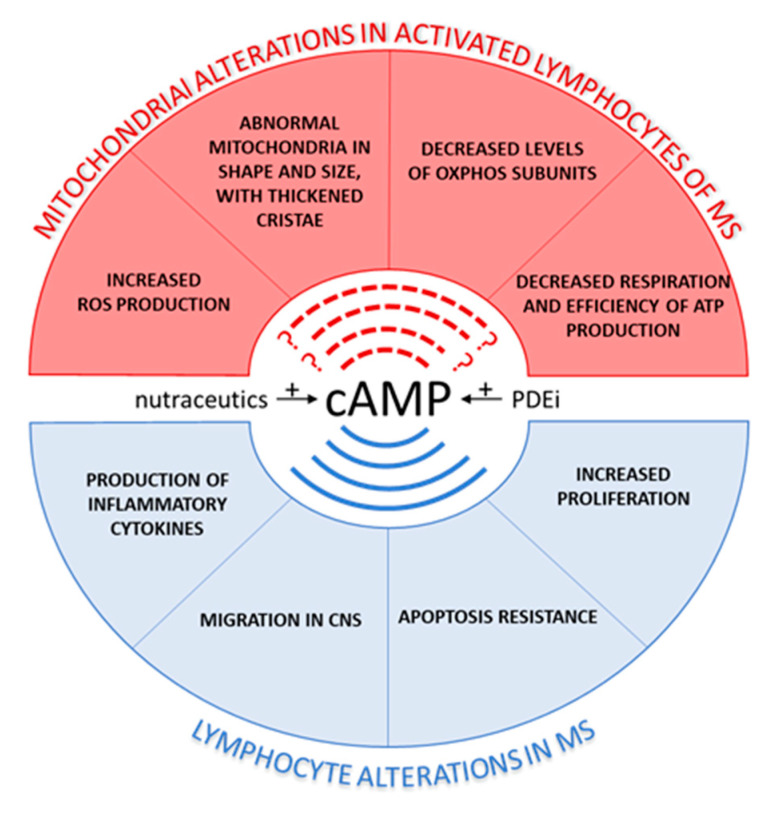
cAMP, the possible crossroad between lymphocytes and mitochondrial dysfunctions in multiple sclerosis (MS). The figure shows the alterations of lymphocytes (blue semicircle) and mitochondrial lymphocytes (red semicircle) observed in MS. cyclic AMP (cAMP) level has been found decrease in peripheral blood mononuclear cells of relapsing remitting (RR) MS patients [84]. The general observation on T cells is that increasing cAMP levels, by phosphodiesyterase inhibitors (PDEi) [74,78,79,80] or by nutraceutics interventions [113], attenuates the T lymphocyte mediated immune response by decreasing the pro-inflammatory cytokines production (e.g., interferon-γ (IFN-γ), tumor necrosis α (TNF-α), and interleukin-1β), T cell proliferation, activation, and migration in central nervous system (CNS) as well as increases their apoptosis. Mitochondrial alterations have been observed in lymphocytes of MS in terms of mitochondrial respiration, coupling, dynamics, structure, biogenesis, and mitochondria-mediated apoptosis. Although cAMP is a master regulator of mitochondrial metabolism and structure [116], no information is available on mitochondrial rescues after increasing cAMP level in MS lymphocytes.

Various nutraceutics, such as curcumin, flavonoids, melatonin, hydroxytirosol (HT), and vitamin D, have been trialed for their efficacy as adjunctive treatments in MS [136]. Beyond their own antioxidant effect, they can have molecular targets implicated in MS pathology, such as inflammation, oxidative stress, mitochondrial dysfunction, and cAMP signaling (Figure 5).

#### 6.2.1. Curcumin

Curcumin is a natural antioxidant polyphenolic compound with anti-inflammatory and immunomodulatory properties, which can influence the immune cell activation, including T cells. Furthermore, curcumin can inhibit the pro-inflammatory cytokines and chemokines expressions by suppressing the nuclear factor kappa-light-chain-enhancer of activated B cells (NF-κB) signaling pathway [137]. In an EAE rat model, the daily treatment with 50 or 100 µg curcumin decreased the pathological and clinical disease severity [138]. In addition, curcumin inhibits cytokine production and phytohemagglutinin-induced lymphocyte proliferation by inhibiting lipopolysaccharide-induced NF-kB target genes [139]. Curcumin exerts various other pharmacological effects, including proapoptotic and anti-proliferative effects [140]. In HT-29 cells, the curcumin-dependent apoptosis includes the activation of caspase and mitochondrial dysfunction primed by enhanced Bax activation [139,141]. In addition, it is reported the involvement of endoplasmic reticulum-stress in curcumin-dependent apoptosis of HL-60 cells [142] and the induction of unfolded protein response (UPR) components promoting apoptosis after the curcumin treatment in activated human CD4^+^ T cells promoting apoptosis [140]. It is also observed that, with the treatment of HEK-293T cells at 10 µM concentration, curcumin upregulated cAMP signaling [143].

#### 6.2.2. Flavonoids

Flavonoids play an important role in the management of MS [144,145]. Flavonoids such as quercetin and epigallocatechin-3-gallate (EGCG) can decrease the production of pro-inflammatory cytokines, including IL-1β, IL-6, and TNF-α [146]. The mechanism of flavonoid action includes the free radical scavenging accompanied with the silencing the pro-oxidative enzymes associated with free radical generation, such as protein kinase C, xanthine oxidase, cyclooxygenase, lipoxygenase, and NADPH oxidase and chelating metals [147]. EGCG is one of the best studied flavonoids in EAE and MS is. It is an active ingredient of green tea and it has been shown to inhibit the differentiation of immature CD4^+^ T cell into Th1 and Th17 effector subsets influencing the immune and inflammation profiles of CNS and lymphoid tissues. This results in a reduction of pro-inflammatory cytokine production, autoreactive T-cell proliferation and Th1 and Th17 subpopulations, and in the increase of regulatory T cell populations [148]. Moreover, in EAE oral-applied, EGCG decreased inflammation by inhibiting TNF-α synthesis in T cells. These results suggest a potential role for EGCG in the prevention and treatment of T-cell-mediated autoimmune diseases [148]. ECGC rat treatment has been found to promote an increase of mitochondrial membrane potential in lymphocytes [149], and mitochondrial DNA content as well as nuclear encoded proteins involved in mitochondrial biogenesis in lymphoblastoide cell cultures [150]. It is also known that EGCG can increase the cAMP cellular level [151,152] by inhibiting PDEs [147] and/or activating adenylyl cyclase [153].

Quercetin is a flavonoid phytoestrogen showing anticancer and anti-inflammatory activities. It is reported that quercetin in EAE model, inhibited IL-12 production and neural antigen-specific Th1 differentiation [154]. Moreover, in jurkat cells, it has been found that quercetin induced apoptosis by activating Bak dependent mitochondrial pathway [155]. It should be noted that quercetin is also an inhibitor of PDE promoting cAMP accumulation [156].

#### 6.2.3. Resveratrol

Resveratrol is a natural polyphenol that is found in more than 70 plant species such as peanuts, cranberry and red grapes. Resveratrol has been shown to display anti-inflammatory effects through a series of mechanisms, including inhibition of the synthesis and release of pro-inflammatory molecules and promotion of apoptosis in T cells [157]. At molecular level, it has been suggested that resveratrol acts by inhibiting PDE [158] and thus increasing the cAMP level culminating in an increase of sirtuin 1 (SIRT1) and PGC1α protein activities which prevents axonal degeneration [159]. Moreover, resveratrol has been observed to induce the amelioration of mitochondrial activity in some neurodegenerative diseases [160,161].

#### 6.2.4. Hydroxytyrosol

HT is one of polyphenolic compounds present in extra virgin olive oil (EVOO). EVOO represents a functional food for prevention of immune and inflammatory disease [162]. This can be due to the capacity of HT to cross the BBB explaining thus its neuroprotective role [163]. It has been proposed that the biological activity of HT can come from its metabolites suggesting that hydroxytyrosol-3-O-glucuronide exhibits a stronger antioxidant activity than hydroxytyrosol itself [164]. The plasma concentration of HT does not only come from the absorption of olive oil polyphenols, but HT also is a by-product of dopamine oxidative metabolism [165]. HT is one of the polyphenols of EVOO with the most powerful anti-inflammatory properties such as reduced secretion of pro-inflammatory cytokines (interleukin (IL)-1α, IL-1β, IL-6, IL-12), tumor necrosis factor-α (TNF-α), and chemokines, inhibition of PGE2 and NO production, and decreased gene expression of iNOS [166]. The beneficial effect of EVOO, and in particular of two of its components, HT and oleic acid, on the improvement of OS in the blood and CNS of EAE rats has been observed [167]. The study reported in the CNS and blood of EAE rats a reduction of lipid peroxidation and protein carbonylation [167]. Interestingly, it is reported that high doses of hydroxytyrosol can induce apoptosis by increasing the expression of caspase 3 gene and the BAX/BCL2 ratio [168]. In different cell lines, in the range of concentration of 0.1–10 µM, HT promotes the activity and expression of PGC1α, which, in turn, promotes the expression and activity of mitochondrial respiratory chain complexes I, II, III, IV, and complex V thus ameliorating mitochondrial function [169,170]. In addition, in serum-starved fibroblast cultures, HT prevents the decline in the expression of the PGC-1α transcription cascade and this effect appears to be associated with the PKA activation and, thus, CREB phosphorylation [171]. In addition to the role of HT on mitochondrial function and biogenesis, studies performed in vivo and in vitro show that HT can control the expression of mitochondrial shaping proteins [172].

#### 6.2.5. Vitamin D

Vitamin D is a steroidal compound metabolized in the liver, skin, epithelial cells, immune cells, and kidneys [173]. Two forms of vitamin D exist, vitamin D2 (ergocalciferol) and vitamin D3 (cholecalciferol). The vitamin D active form is 1,25-dihydroxycholecalciferol (1,25-(OH)2D3) derived by two-step transformation process catalyzed by 25-hydroxylase-CYP2R1 and 1-alpha-hydroxylase. The principal organs involved in vitamin D metabolism are liver and kidney, however, other tissues such as immune cells, parathyroid gland and epithelial cells perform the metabolic conversion in vitamin D active form [173]. Vitamin D3 is primarily found in fatty animal-sourced foods, such as egg yolk and fish oil, while vitamin D2 in plant-sourced foods, for instance fortified foods and UV light-grown mushrooms [174]. The vitamin D receptors are present in most cells, like activated monocytes, B and T cells, and microglia and tissues like skin and intestine. Very interestingly, vitamin D supplementation as well as a higher circulating vitamin D level are associated with a reduced risk of MS [175]. Vitamin D has also been observed to have immunomodulatory effects, increasing Th2 and Tregs activity and decreasing Th1 activity, and thus it may play a role in the MS etiology [175]. The active vitamin D regulates the expression of iNOS and the production of NO in different cells, such as astrocytes, microglial cells, and macrophages [176]. The protective mechanism against EAE involves a decreased accumulation of T cells and macrophages in the CNS [177,178], an increased apoptosis of inflammatory cells, and enhanced survival of CNS cells. However, the activated inflammatory cells are able to produce 1,25-(OH)2D3, that subsequently exerts anti-inflammatory activities against these cells [179]. In vivo molecular experiments show that 1,25-(OH)2D3 directly acts on pathogenic CD4^+^ T cells and inhibits EAE via receptors of vitamin D in T lymphocytes [180]. It should be noted that 1,25-(OH)2D3 can suppress lymphocyte mitogenesis by acting synergistically with agents that augment cellular cAMP level [180]. Interestingly, vitamin D supplementation ameliorates mitochondrial cristae shape by modulating mitofusin-1/2 (Mfn1/2), OPA1 and DRP1 expression [181].

#### 6.2.6. Melatonin

Melatonin is produced by the pineal gland in response to the darkness during the night. Melatonin is also found in several foods, such as fruits, vegetables, seeds, and herbs [182]. The level of melatonin has been found impaired in MS patients, and exogenous melatonin administration improves the disease by modulating the Th1/Th17/Treg responses in MS animal models [183] and ameliorates several symptoms and quality of life in MS patients [184]. In addition, melatonin has a cytoprotective actions by controlling OS, mitochondrial homeostasis, and apoptosis [185]. It exerts its actions by binding to G-protein-coupled receptors at the membrane and by interacting with intracellular targets to control signal transduction pathways, redox processes, and free radicals scavenging [186]. Melatonin stimulates the activities of glutathione peroxidase and SOD but inhibits the prooxidant enzyme NOS, thus it may be a potent antioxidant molecule acting also at mitochondrial level. Indeed, in several cell types, melatonin has been found to increase the activities of respiratory chain complexes I and IV, stimulate oxidative phosphorylation, and promote a rise in ATP level [187]. Furthermore, melatonin improves mitochondrial dynamics by reducing DRP1 expression and increasing that of fusion proteins, Mfn2 and OPA1 [188].

## 7. Conclusions

In this review we have highlighted the involvement of mitochondria, ROS production, cAMP pathway, apoptosis, and their interconnection in alteration of T lymphocytes homeostasis in MS. So far, in lymphocytes of MS patients, a reduced activity of mitochondrial enzymes and a bioenergetic decoupling of mitochondria in term of efficiency of ATP production associated with increased production of anion superoxide have been observed. At the same time, the increase of cAMP, that reduced T cell proliferation, strongly modulates mitochondrial activity in terms of mitochondrial respiration, coupling, dynamics, structure, biogenesis, and mitochondria-mediated apoptosis (Figure 5). Nutritional status in MS patients and dietary supplementation have been proposed as potential critical factors that can affect risk and progression of the disease [189] in fact, several dietary supplements might reduce inflammation and fatigue and increase autoimmunity tolerance in patients with MS, even if it should be noted that dietary supplementation is not an effective clinical indication as complementary treatment for MS patients. Further research on nutraceutics and their mechanisms and molecular pathways involved should be performed in order to verify the link among inflammation, cAMP, and mitochondria. So, an increasing cAMP level, by inhibiting specific PDEs and/or by using nutraceutics, can be considered as a possible strategy to limit the activation of T cells.

## Figures and Tables

**Table 1:** Proteins involved in apoptosis resistance of lymphocytes in multiple sclerosis.

Protein	Deregulation in MS	Reference
Bcl2	Increased expression	[29,89,90]
OPA1	Altered proteolytic processing	[25,98]
PHB2	Increased expression	[25,106,107]
SIRT3	No response to oxidative stress	[25]
OMA1	No response to oxidative stress	[25]
ATG5	Upregulation and post-translational modification	[112]
